# Closed Traumatic Laryngeal Injury With Partial Tracheal Rupture: A Case Report and Literature Review

**DOI:** 10.7759/cureus.107084

**Published:** 2026-04-15

**Authors:** Zefeng Li, Yixin Bai, Xianbai Zhang, Peng Ni, Changhong Gu

**Affiliations:** 1 Department of Otolaryngology-Head and Neck Surgery, Dandong Central Hospital, Dandong, CHN

**Keywords:** airway injury, blunt neck trauma, emergency tracheostomy, laryngotracheal trauma, partial tracheal rupture

## Abstract

Blunt neck trauma may cause major laryngotracheal injury, a rare but potentially life-threatening condition in which early airway decision-making is critical. This report aims to describe the clinical course of a patient with closed traumatic laryngeal injury with partial tracheal rupture and to propose a practical airway management algorithm for similar high-risk scenarios.

A 61-year-old man sustained blunt head, neck, and chest trauma after a ground-level fall. He presented with hoarseness, intermittent hemoptysis, mild mixed dyspnea, and extensive subcutaneous emphysema of the neck and chest. Computed tomography (CT) demonstrated subglottic tracheal deformity/narrowing, suspected cricoid cartilage fracture, and pneumomediastinum, raising concern for major laryngotracheal injury with partial tracheal rupture. Because of the anticipated difficulty and hazards of conventional endotracheal intubation in distorted airway anatomy, emergency tracheostomy was performed under local anesthesia. This was followed by staged tracheoplasty with placement of a silicone laryngotracheal mold. The patient was successfully decannulated at two weeks. On postoperative day 18, dynamic flexible laryngoscopy demonstrated reduced bilateral vocal fold mobility with incomplete glottic closure, while the airway remained patent and the patient had no respiratory compromise.

This case highlights the importance of early recognition of major airway injury after blunt neck trauma, prompt multidisciplinary assessment, and timely establishment of a surgical airway when oral intubation is predicted to be unsafe. Based on this experience, we propose a practical airway management algorithm that emphasizes early transition to a surgical airway and definitive reconstruction in selected patients with suspected major laryngotracheal disruption.

## Introduction

Laryngotracheal trauma (LTT) is an uncommon but potentially life-threatening injury, often encountered in the setting of polytrauma. Owing to its rarity and sometimes subtle presentation, LTT may be underrecognized, leading to delayed diagnosis and treatment [1]. The severity of symptoms is not always proportional to the extent of injury [2]. The spectrum of LTT ranges from intralaryngeal hematomas to tracheal rupture and, in the most severe cases, complete transection [1].

LTT accounts for an estimated 1% of all traumatic injuries, with an incidence of approximately one in 30,000 cases, and is more common in adults, who comprise over 75% of acute fatalities [3]. Typical symptoms include hoarseness, hemoptysis, neck pain, dysphonia, dyspnea, dysphagia, and odynophagia [4]. Physical findings may include ecchymosis, edema, neck and laryngeal tenderness, loss of the laryngeal prominence, and subcutaneous emphysema [5].

Major laryngotracheal injuries, including partial tracheal rupture, are among the most challenging conditions to manage and rehabilitate because of the complexity of their mechanisms of injury and the intricacies of airway reconstruction [1]. Research on this entity is hampered by small, heterogeneous samples and incomplete data [6].

This report aims to describe the diagnostic and therapeutic course of a patient with closed traumatic laryngeal injury and partial tracheal rupture and to propose a practical airway management algorithm for suspected major laryngotracheal injury after blunt neck trauma.

## Case presentation

A 61-year-old man, weighing 100 kg and with a known history of cardiac arrhythmia, was transferred to the emergency department of Dandong Central Hospital six hours after sustaining head, neck, and chest trauma from an accidental fall. Urgent computed tomography (CT) of the head, neck, and chest was performed shortly after arrival, followed by immediate multidisciplinary consultations (thoracic surgery, orthopedics, neurosurgery, and otolaryngology-head and neck surgery (ENT-HNS)). On arrival, he was fully alert and appeared to have difficulty maintaining a comfortable posture.

Past medical and social history

History was obtained from the family members. The patient had a long-standing history of cardiac arrhythmia. He had no known history of diabetes mellitus, hypertension, hepatitis, tuberculosis, or other communicable diseases. He had undergone an appendectomy previously and had no history of blood transfusion. He reported long-term tobacco use but denied alcohol consumption, occupational toxin/dust exposure, or radiation exposure. No food or drug allergies were reported. Family history was unremarkable.

Physical Examination

The patient appeared distressed and adopted a forced posture. He was fully alert and cooperative; the history was obtained from family members. Vital signs were as follows: temperature: 36.5°C; heart rate: 104 beats/min; respiratory rate: 24 breaths/min; and blood pressure: 135/86 mmHg. He had hoarseness with intermittent hemoptysis and mild mixed dyspnea at rest. The anterior neck was markedly tender on palpation. Palpation also revealed extensive subcutaneous crepitus over the neck and upper chest. Breath sounds were bilaterally decreased.

Differential Diagnosis

Given the blunt cervicothoracic trauma with hoarseness, hemoptysis, dyspnea, and extensive subcutaneous emphysema, the differential diagnosis included laryngotracheal injury (including tracheal rupture), laryngeal fracture, esophageal perforation, pneumothorax, pulmonary contusion, and major vascular injury of the neck/mediastinum.

Diagnostic Assessment and Investigations

Preoperative flexible laryngoscopy was not performed because the patient required urgent evaluation and airway management, leaving insufficient time to complete laryngoscopic examination. Therefore, diagnostic assessment relied on clinical findings and urgent CT of the head, neck, and chest.

**Figure 1:**
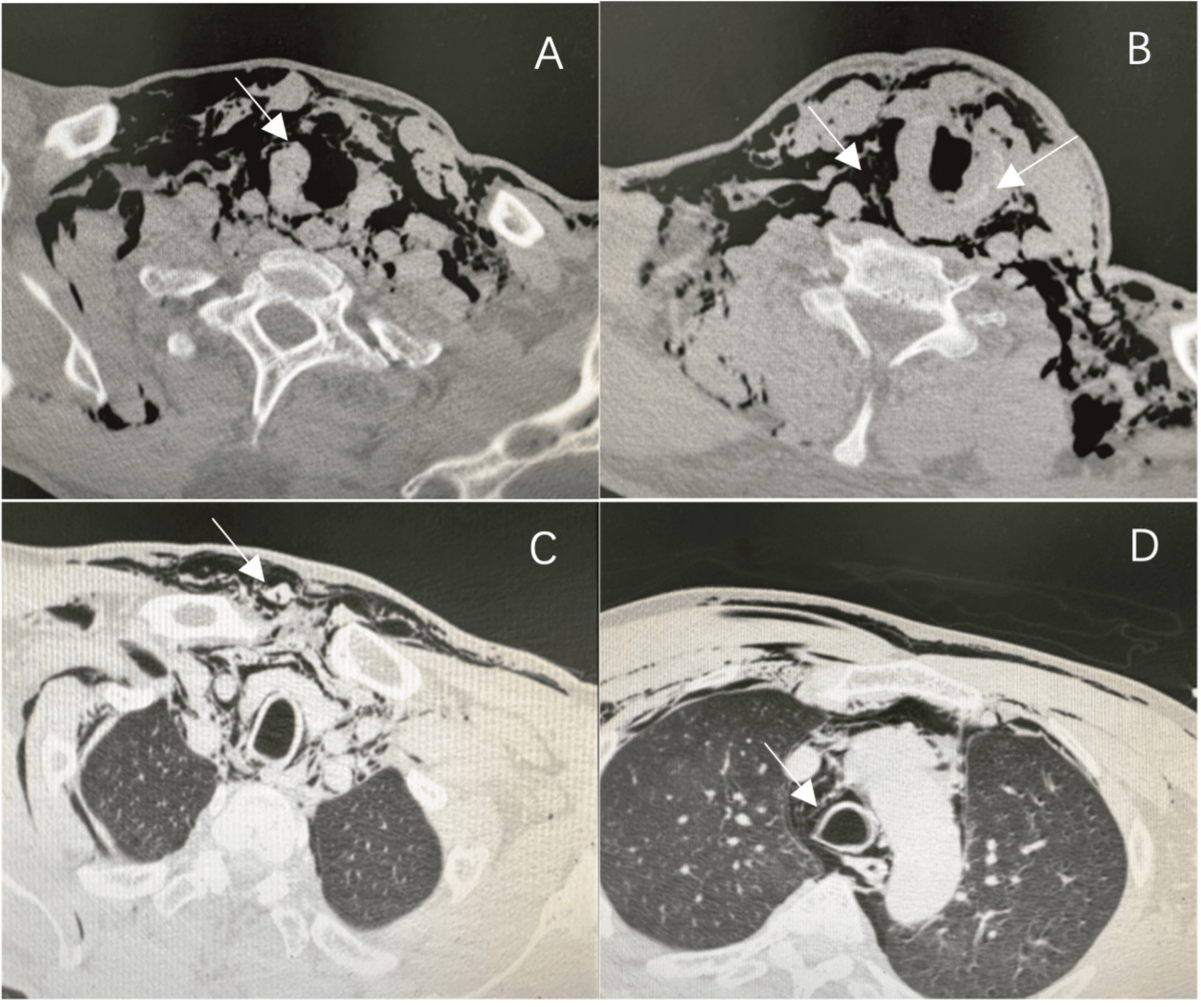
Preoperative CT imaging of the neck and chest (A) Airway CT demonstrating an irregular contour and deformity of the subglottic tracheal lumen. (B) CT showed a linear lucency through the cricoid cartilage, suggestive of cartilage fracture, with extensive subcutaneous emphysema in the cervical soft tissues. (C) Chest CT showing subcutaneous emphysema along the anterior chest wall. (D) Chest CT demonstrating pneumomediastinum with free air tracking along the mediastinum

CT of the neck demonstrated an irregular contour of the subglottic trachea with luminal narrowing (Figure 1A) and a linear lucency across the cricoid cartilage suggestive of fracture (Figure 1B). Extensive subcutaneous emphysema was present in the cervical soft tissues (Figure 1B) and anterior chest wall (Figure 1C), along with pneumomediastinum (Figure 1D).

At initial presentation, the diagnosis of major laryngotracheal injury was based on the clinical pattern of blunt cervicothoracic trauma with hoarseness, hemoptysis, dyspnea, and extensive subcutaneous emphysema. CT further increased suspicion by demonstrating subglottic airway deformity, suspected cricoid cartilage fracture, and pneumomediastinum. The full extent of laryngotracheal framework disruption was subsequently confirmed intraoperatively.

The patient was initially admitted under thoracic surgery because of concomitant chest trauma and extensive cervicothoracic emphysema, and he received electrocardiographic monitoring, supplemental oxygen, and nasogastric tube placement. ENT-HNS was consulted emergently for suspected major laryngotracheal injury and assumed responsibility for airway decision-making.

Given the mechanism of injury and clinical findings, urgent multidisciplinary consultations were obtained from orthopedic surgery, neurosurgery, and ENT-HNS. The orthopedic and neurosurgical teams found no indication for immediate surgical intervention and recommended supportive care, close monitoring, and further investigations. In contrast, the ENT-HNS team strongly advocated for an emergency tracheostomy because of the suspected laryngotracheal injury and the anticipated difficulty and risk associated with conventional airway management.

An emergency tracheostomy was performed due to the substantial risks associated with awake intubation in this setting [7]. These risks included the potential for severe coughing triggered by sedation and topical anesthesia, leading to increased airway bleeding and secretions, thereby worsening airway obstruction in a vicious cycle [8]. Furthermore, the accumulation of blood and secretions could impair the efficacy of topical anesthetics, while deeper sedation might suppress protective airway reflexes [9]. Persistent tracheal hemorrhage was also a concern, as it could obscure visualization during fiberoptic bronchoscopy, which is limited by a narrow suction channel [10]. Although the patient’s vital signs were initially stable, he was at high risk of rapid deterioration [11]. Any time-consuming airway maneuver in this context could have resulted in catastrophic decompensation [1].

Emergency Tracheostomy (January 16, 2023)

Emergency tracheostomy was performed under local infiltration anesthesia (1% lidocaine with a small amount of epinephrine). A midline incision above the sternal notch was made; the 3rd-4th tracheal rings were exposed and opened; an 8.5-cm plastic tracheostomy tube was inserted. Estimated blood loss was approximately 2 mL, and no intraoperative complications were recorded. Following the emergency tracheostomy, the patient was transferred to the ENT-HNS department for a planned second‑stage procedure consisting of tracheoplasty and placement of a laryngotracheal mold.

Staged Tracheoplasty and Laryngotracheal Mold Placement (January 31, 2023)

Under general anesthesia, staged reconstruction was performed via a U-shaped anterior neck incision with subplatysmal flap elevation. Operative findings described severe injury involving the laryngeal framework and upper trachea, including markedly fractured/deformed cricoid cartilage and damage/occlusion of the 1st-3rd tracheal rings with loss of a patent lumen.

**Figure 2:**
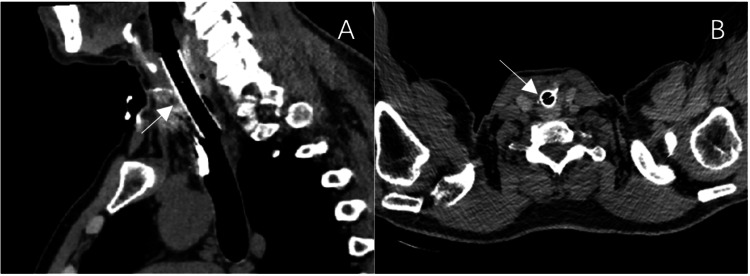
Postoperative CT imaging of the reconstructed airway (A) Three-dimensional CT reconstruction obtained on postoperative day 8 demonstrating appropriate positioning of the tracheostomy tube within the reconstructed tracheal lumen and a patent airway. (B) Three-dimensional CT reconstruction obtained on postoperative day 14 demonstrating the laryngotracheal mold in a satisfactory position without evidence of displacement.

A longitudinal incision was made to connect proximal and distal airway lumens; granulation-like tissue was removed; injured cartilage was repositioned as feasible; hemostasis and irrigation were performed. A 2.0 cm silicone laryngotracheal mold was placed. With suspension laryngoscopy, the mold’s upper end was positioned at the subglottis and secured using a wire fixation technique. An anterior airway wall defect (approximately 3.5 × 1.0 cm) was repaired using an inferiorly based strap-muscle fascia flap with the fascia oriented toward the mold. A left cervical drain was placed, and an 8.5 cm cuffed tracheostomy tube was inserted. Estimated blood loss was ~50 mL, with no documented intraoperative complications. Postoperative airway CT with three-dimensional reconstruction obtained on postoperative day (POD) 8 confirmed appropriate positioning of the tracheostomy tube within the reconstructed tracheal lumen and demonstrated a patent airway (Figure 2A).

**Figure 3:**
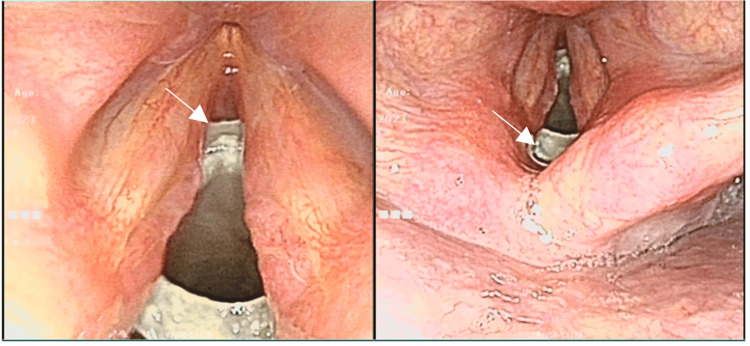
Postoperative flexible laryngoscopy showing incomplete glottic closure with a patent airway Flexible laryngoscopy was performed on postoperative day 18 after decannulation, with the laryngotracheal mold in situ. The still image shows incomplete glottic closure (glottic insufficiency), while the airway remains patent. Dynamic examination at the time of laryngoscopy demonstrated reduced bilateral vocal fold mobility

At two weeks postoperatively (POD 14), the tracheostomy tube was removed while the laryngotracheal mold was left in situ. Repeat airway CT with three-dimensional reconstruction demonstrated the mold in a satisfactory position without evidence of displacement (Figure 2B). The patient exhibited no signs of respiratory distress. Dynamic flexible laryngoscopy was subsequently performed on POD 18 after decannulation and demonstrated reduced bilateral vocal fold mobility with incomplete glottic closure, while the airway remained patent (Figure 3). The timeline of the patient’s clinical course and major interventions is summarized in Table 1.

**Table 1:** Timeline of clinical course and interventions SAH: subarachnoid hemorrhage; EBL: estimated blood loss; POD: postoperative day

Date/Time	Event
2023-01-16 (injury)	Ground-level fall causing blunt head/neck/chest trauma
2023-01-16 16:31	Admission to Dandong Central Hospital
2023-01-16 16:35	Initial assessment; vitals T36.5, P104, R24, BP135/86
2023-01-16 17:32	Multidisciplinary consults; ENT recommends emergency tracheostomy
2023-01-16 19:14	CT neck/chest/head suggests airway injury + pneumomediastinum; traumatic SAH
2023-01-16 20:35–21:05	Tracheostomy under local infiltration anesthesia; tube 8.5 cm; EBL ~2 mL
2023-01-31 16:00–17:45	Tracheoplasty + mold placement under general anesthesia; EBL ~50 mL
2023-02-08	Replacement of the tracheostomy tube with a titanium tracheostomy tube
2023-02-11	Tracheostomy tube capping with phonation training
2023-02-14	Removal of the tracheostomy tube
2023-02-18	(POD 18) Dynamic flexible laryngoscopy after decannulation showed reduced bilateral vocal fold mobility and incomplete glottic closure; airway patent; hoarseness persisted
2023-02-18	Discharged; outpatient follow-up arranged

Hospital course and outcomes

The patient did not require ICU admission and was managed on the ward with close airway monitoring. Supplemental oxygen was delivered via blow-by oxygen to the tracheostomy as needed. No airway bleeding or infectious complications occurred during hospitalization. The subcutaneous emphysema and pneumomediastinum gradually decreased and resolved during hospitalization.

Perioperative medical management included cefuroxime sodium, dexamethasone, and nebulized budesonide; pain was well-controlled without analgesics. Swallowing and oral intake were advanced in a stepwise manner: on POD 11 (POD was calculated from the date of tracheoplasty with mold placement, January 31, 2023), capping trials were initiated with phonation training; on POD 12, oral intake was started but was complicated by coughing/choking. The tracheostomy tube was removed on POD 14. By POD 17, oral intake was tolerated without coughing/choking, and the nasogastric tube was removed.

Before discharge (POD 18, after decannulation), dynamic flexible laryngoscopy (with the laryngotracheal mold in situ) demonstrated reduced bilateral vocal fold mobility with incomplete glottic closure during phonation, while the airway remained patent (Figure 3). The still image illustrates incomplete glottic closure at the time of examination. At discharge (POD 18), the patient had no dyspnea; however, hoarseness persisted. Swallowing function was acceptable, and the incision was well-healed. Outpatient follow-up was scheduled; however, the patient did not return, and no post-discharge follow-up data were available.

## Discussion

Closed laryngeal trauma with associated partial tracheal rupture is extremely challenging to manage. Universally accepted guidelines are lacking, largely because injury mechanisms vary widely, and airway reconstruction is technically complex [11]. Based on the Schaefer-Fuhrman classification, this case was most consistent with a high-grade laryngeal injury (approximately Grade IV), given the suspected cricoid fracture, marked subglottic airway deformity/stenosis on CT, extensive cervicothoracic subcutaneous emphysema and pneumomediastinum, and intraoperative evidence of severe laryngotracheal framework disruption [12]. These high-risk features suggested potential airway instability and a high likelihood of failed or hazardous endotracheal intubation; therefore, an emergency tracheostomy was prioritized as the initial airway strategy [1].

Airway management decision-making

The patient’s constellation of symptoms, such as hoarseness, hemoptysis, dyspnea, and subcutaneous emphysema, strongly suggested a significant airway injury [3]. Distortion of the normal cervical anatomical landmarks following trauma rendered endotracheal intubation not only technically difficult but also potentially dangerous, with a risk of creating false passages or worsening the existing injury [1].

Rapid sequence induction (RSI) is commonly used when upper airway landmarks are preserved, and oxygenation is relatively secure [13]. However, in patients with a predicted difficult airway (e.g., Mallampati class ≥ III, mouth opening < 3 cm, or suspected airway disruption), RSI may lead to a “cannot intubate, cannot oxygenate” (CICO) situation and complete loss of airway control [13].

Awake intubation is traditionally considered safer in anticipated difficult-airway scenarios, but in this case, it was deemed too risky [13]. Severe coughing triggered by topical anesthesia or sedation could exacerbate bleeding, increase secretions, and obstruct the glottic view, thereby delaying definitive airway control and increasing the risk of complete airway loss [1].

Role of surgical airway

In this case, the scenario was effectively evolving into a CICO situation, and a surgical airway was rapidly selected as the definitive strategy [11]. Although cricothyrotomy is generally faster and associated with a lower immediate complication rate in emergency settings, tracheostomy was preferred here because of the closed neck trauma and the potential presence of fractures of the thyroid and cricoid cartilages [14].

The “three-in-one progressive strategy” for difficult airway management suggests sequential attempts with a face mask airway, oral intubation, and supraglottic devices [13]. If all three approaches fail or are deemed unsafe, an emergency surgical airway must be established without delay [15]. Each unsuccessful attempt consumes valuable time and reduces the already narrow safety window for successful intervention, with an exponential increase in the risk of hypoxic injury and death [13].

**Figure 4:**
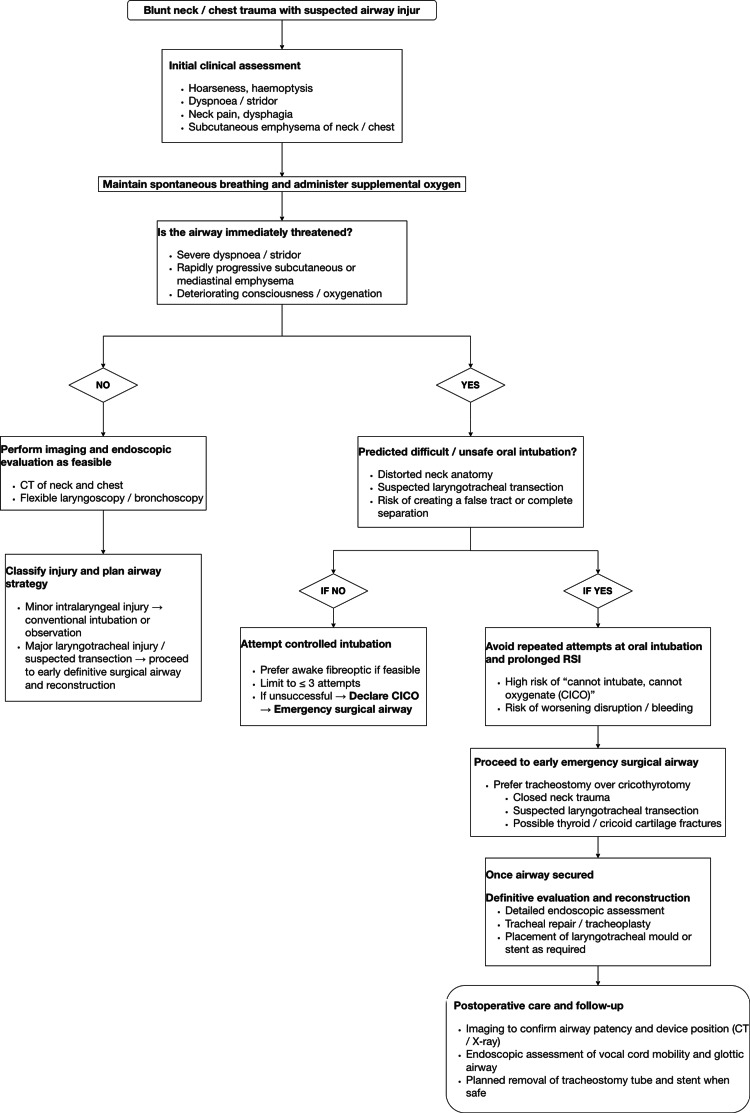
Airway management algorithm for patients with suspected laryngotracheal injury after blunt neck trauma Proposed practical algorithm for airway management in patients with suspected major laryngotracheal injury, including partial tracheal rupture, after blunt neck trauma. This algorithm is intended as a pragmatic, case-informed framework based on this report and selected literature, rather than a validated protocol. Initial assessment focuses on early recognition of signs suggesting a threatened airway, such as hoarseness, hemoptysis, dyspnea, subcutaneous emphysema, and radiologic evidence of airway disruption. In patients in whom oral intubation is predicted to be difficult or unsafe, repeated attempts at conventional intubation or prolonged rapid sequence induction should be avoided. Instead, early emergency surgical airway access (preferably tracheostomy in the setting of closed neck trauma and suspected laryngotracheal disruption) is recommended, followed by definitive evaluation, reconstruction, and structured postoperative follow-up

Based on this case and the relevant literature, we propose a practical decision-making algorithm for airway management in suspected major laryngotracheal injury, including partial tracheal rupture, after blunt neck trauma (Figure 4). The algorithm emphasizes early recognition of a threatened airway, prompt identification of patients in whom oral intubation is predicted to be unsafe, avoidance of repeated attempts at conventional intubation or prolonged rapid sequence induction when airway disruption is suspected, and timely transition to an emergency surgical airway followed by definitive evaluation and reconstruction when indicated [1].

Time management and operational strategy

Difficult airway guidelines typically recommend no more than three attempts at intubation before abandoning a particular approach [16]. Rigid adherence to this principle and strict control of repeat attempts are essential to minimize time delays and avoid “operational lock,” a situation in which the team becomes fixated on repeated unsuccessful techniques [13].

In the present case, any intubation attempt was effectively “racing” against the progression of pneumomediastinum and cardiorespiratory compromise. The worst-case outcomes, including cerebral hypoxia and death, had to be actively considered [17]. In this context, early abandonment of repeated attempts at oral intubation in favor of a surgical airway was, in our judgment, the most appropriate strategy for this patient.

Fiberoptic bronchoscopy considerations

Oral intubation under fiberoptic bronchoscope (FOB) guidance can, in theory, help identify the exact site of airway disruption and direct the endotracheal tube distal to the injury, thereby avoiding false tracts [11]. However, such an approach requires a “failure-oriented” mindset, recognizing the high likelihood of poor visualization in the presence of active bleeding and heavy secretions, even for experienced operators [18].

In this case, FOB was available and initially considered. However, the anticipated time required for the procedure, the difficulty in clearing blood and clots, and the challenges of identifying the level of tracheal rupture in a disrupted, bleeding airway made this option less favorable [1]. The potential delay in securing a definitive airway posed an unacceptable risk [11].

Our decision does not negate the role of FOB in urgent, difficult airway management [19]. Rather, it reflects a pragmatic choice tailored to this patient’s condition, balancing theoretical benefits against the realities of time pressure, bleeding, and anatomical disruption.

Multidisciplinary collaboration and training

We believe that the confidence and technical proficiency of the surgical team, particularly the ENT surgeon, played a pivotal role in the favorable outcome of this case. This confidence was largely attributable to ongoing structured training in difficult airway management and regular simulation-based drills aimed at developing “muscle memory” for high-stress procedures [13].

Real-time technical support from the multidisciplinary team during the tracheostomy was crucial. Despite the challenging anatomy and the risk of airway bleeding, the airway was secured promptly; the site of disruption was rapidly identified and managed. This experience underscores the importance of scenario-based, high-fidelity simulation training in developing operational readiness for time-critical interventions such as emergency surgical airway access [20].

Outcome and imaging correlation

The satisfactory postoperative imaging findings (Figures 2A-2B), together with a patent airway on POD 18 flexible laryngoscopy, underscore the potential for good short-term functional recovery when early definitive reconstruction is undertaken after timely surgical airway establishment [1]. Dynamic laryngoscopy demonstrated reduced bilateral vocal fold mobility, and the still image showed incomplete glottic closure during the molding period; these findings may reflect posttraumatic edema, laryngeal framework injury, and/or mechanical restriction related to the mold [11].

Strengths and limitations

This case highlights a rapid, pragmatic airway strategy in suspected major laryngotracheal injury after blunt neck trauma. A key strength was early recognition of high-risk features (hoarseness, hemoptysis, dyspnea, and extensive subcutaneous emphysema and pneumomediastinum) and prompt multidisciplinary decision-making, which supported the timely establishment of a surgical airway and avoided potentially hazardous repeated attempts at oral intubation in distorted anatomy [13]. Staged reconstruction with a silicone laryngotracheal mold restored airway patency and enabled early decannulation.

Several limitations should be acknowledged. First, preoperative flexible laryngoscopy/bronchoscopy was not performed because urgent airway control was prioritized, limiting direct preoperative characterization of the injury. Second, this is a single-patient report and therefore cannot define optimal management for all traumatic laryngotracheal disruptions. Third, long-term functional outcomes (airway stability after mold removal, voice quality, and swallowing function) could not be assessed because the patient had no postdischarge follow-up.

## Conclusions

Closed laryngeal trauma with major laryngotracheal injury, including partial tracheal rupture, is rare, potentially life-threatening, and particularly challenging when airway anatomy is disrupted. In selected patients in whom difficult airway management is anticipated and conventional oral intubation is predicted to be unsafe, early and decisive establishment of a surgical airway may be life-saving.

This case demonstrates that a rapid multidisciplinary strategy, strict time management, and a low threshold for early surgical airway intervention can result in successful short-term airway rescue and airway patency. Based on this experience, we propose a practical airway management algorithm for suspected major laryngotracheal injury after blunt neck trauma, emphasizing early recognition of a threatened airway, avoidance of repeated hazardous intubation attempts, timely transition to surgical airway access, and subsequent definitive reconstruction when indicated. Because this is a single case with no postdischarge follow-up, the proposed algorithm should be interpreted as a pragmatic, case-informed suggestion rather than a validated management framework.
